# Marine alkaloid monanchoxymycalin C: a new specific activator of JNK1/2 kinase with anticancer properties

**DOI:** 10.1038/s41598-020-69751-z

**Published:** 2020-08-06

**Authors:** Sergey A. Dyshlovoy, Moritz Kaune, Malte Kriegs, Jessica Hauschild, Tobias Busenbender, Larisa K. Shubina, Tatyana N. Makarieva, Konstantin Hoffer, Carsten Bokemeyer, Markus Graefen, Valentin A. Stonik, Gunhild von Amsberg

**Affiliations:** 1 Department of Oncology, Hematology and Bone Marrow Transplantation with Section Pneumology, Hubertus Wald Tumorzentrum, University Cancer Center Hamburg (UCCH), University Medical Center Hamburg-Eppendorf, Martinistrasse 52, 20251 Hamburg, Germany; 2 G.B. Elyakov Pacific Institute of Bioorganic Chemistry, Far-East Branch, Russian Academy of Sciences, 690022 Vladivostok, Russia; 3 School of Natural Sciences, Far Eastern Federal University, 690091 Vladivostok, Russian Federation; 4 Martini-Klinik, Prostate Cancer Center, University Hospital Hamburg-Eppendorf, Martinistrasse 52, 20251 Hamburg, Germany; 5 Department of Radiotherapy and Radiation Oncology, Hubertus Wald Tumorzentrum, University Cancer Center Hamburg (UCCH), University Medical Center Hamburg-Eppendorf, Martinistrasse 52, 20251 Hamburg, Germany; 6 UCCH Kinomics Core Facility, Hubertus Wald Tumorzentrum, University Cancer Center Hamburg (UCCH), University Medical Center Hamburg-Eppendorf, Martinistrasse 52, 20251 Hamburg, Germany; 7 Laboratory of Experimental Oncology, Department of Oncology, Hematology and Bone Marrow Transplantation with Section Pneumology, Hubertus Wald Tumorzentrum, University Cancer Center Hamburg (UCCH), University Medical Center Hamburg-Eppendorf, Campus Forschung (N27, room 04.082), Martinistrasse 52, 20251 Hamburg, Germany

**Keywords:** Marine biology, Marine chemistry

## Abstract

Monanchoxymycalin C (MomC) is a new marine pentacyclic guanidine alkaloid, recently isolated from marine sponge *Monanchora pulchra* by us. Here, anticancer activity and mechanism of action was investigated for the first time using a human prostate cancer (PCa) model. MomC was active in all PCa cell lines at low micromolar concentrations and induced an unusual caspase-independent, non-apoptotic cell death. Kinase activity screening identified activation of mitogen-activated protein kinase (MAPK) c-Jun N-terminal protein kinase (JNK1/2) to be one of the primary molecular mechanism of MomC anticancer activity. Functional assays demonstrated a specific and selective JNK1/2 activation prior to the induction of other cell death related processes. Inhibition of JNK1/2 by pretreatment with the JNK-inhibitor SP600125 antagonized cytotoxic activity of the marine compound. MomC caused an upregulation of cytotoxic ROS. However, in contrast to other ROS-inducing agents, co-treatment with PARP-inhibitor olaparib revealed antagonistic effects indicating an active PARP to be necessary for MomC activity. Interestingly, although no direct regulation of p38 and ERK1/2 were detected, active p38 kinase was required for MomC efficacy, while the inhibition of ERK1/2 increased its cytotoxicity. In conclusion, MomC shows promising activity against PCa, which is exerted via JNK1/2 activation and non-apoptotic cell death.

## Introduction

Secondary metabolites are natural organic compounds which, unlike primary metabolites, are usually not directly involved in the growth and survival of the individual organism. However, they may contribute to protective or interactive processes. Therefore, these molecules often reveal pronounced bioactivity. Secondary metabolites of marine invertebrates consist of a variety of small molecules belonging to different structural groups^1,2^. Here, pentacyclic guanidine alkaloids represent an important subgroup. A significant number of these compounds has been isolated from marine sponges belonging to the Monanchora family (for review see Refs.^3,4^). They exhibit a broad spectrum of bioactivities, including pronounced anticancer activities^5–9^. However, to date the number of studies analyzing these compounds is still limited, mainly due to the many challenges associated with the collection of deep-sea sponges. Nevertheless, some cellular targets and processes mediating the bioactivity of marine derived pentacyclic guanidine metabolites have been identified^4^. One of the first representatives of this structural group, alkaloid ptilomycalin A was primarily isolated from Caribbean sponge *Ptilocausis spiculifer* as well as from a Red Sea sponge *Hemimycale* sp. in 1989^10^, and later by our group from marine sponge *Monanchora pulchra*, collected in Russian Pacific Far East in 2013^11^. This molecule as well as some related compounds were shown to induce DNA fragmentation and caspase-3/7 activation in cancer cells^9^. Remarkably, pentacyclic alkaloid monanchocidin A^12^ induced cytotoxic autophagy, i.e. type II programmed cell death at low and lysosomal membrane permeabilization at higher concentrations in human germ cell tumor cells enabling the compounds to overcome drug resistance against standard therapies^13^. A global proteomic screening-based analysis revealed anti-migratory activity of monanchocidin A towards human cancer cells^14^.

Structurally related crambescidin alkaloids induced cell cycle arrest of human cancer cells via downregulation of CDK 2/6 and cyclins D/A, as well as simultaneous upregulation of several CDK inhibitors^15,16^. The compounds promoted the differentiation of K562 cells^17^ and blocked several ion channels^18,19^. Crambescidin alkaloids were found to inhibit cancer cell migration via alteration of cytoskeleton dynamics, suppression of cell-to-cell and cell-to-matrix adhesion, as well as inhibition of tight junctions formation^20^.

**Figure 1:**
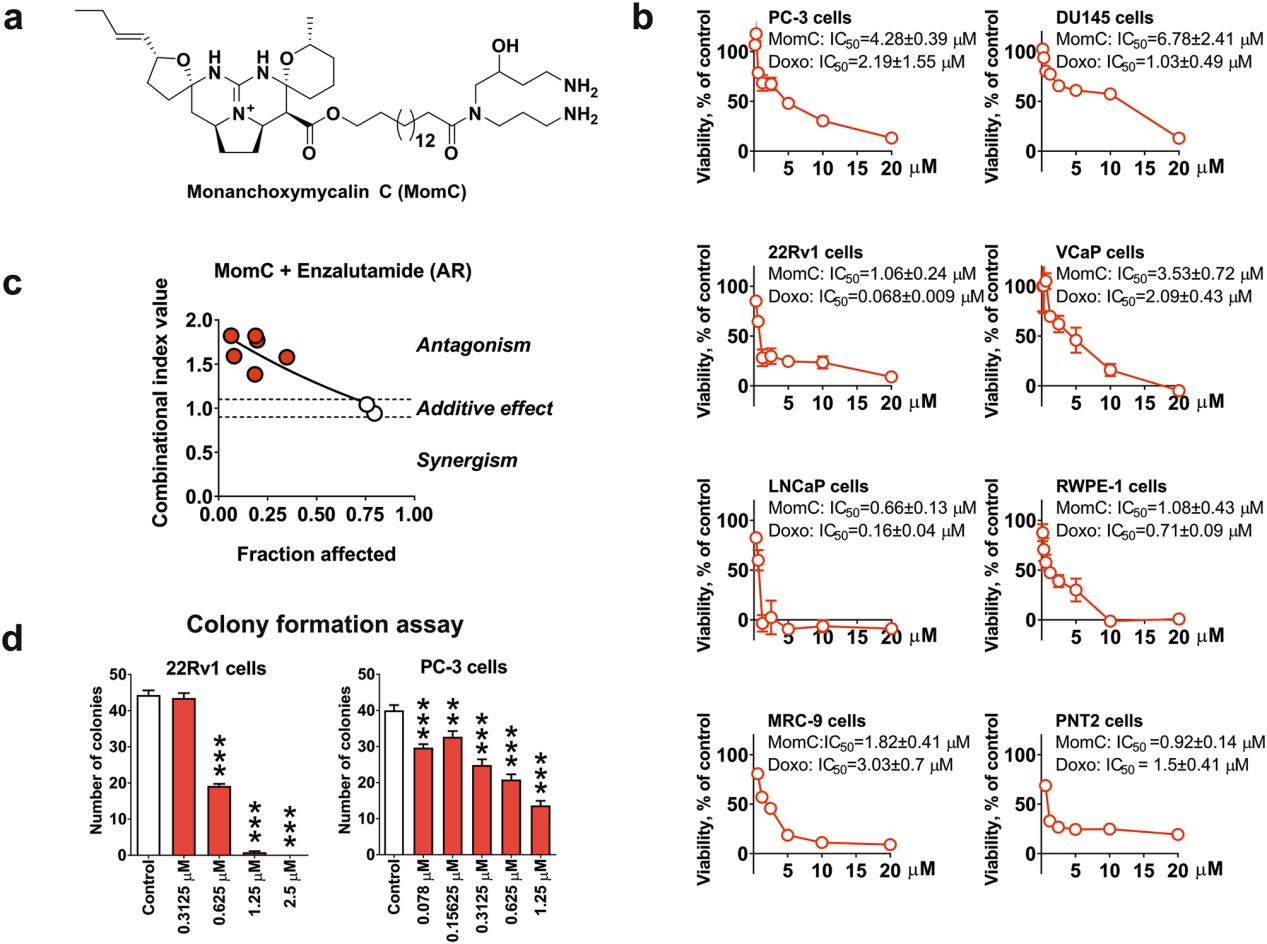
Structure of monanchoxymycalin C (MomC) (**a**). Cytotoxicity profiles of MomC in different human prostate cancer and non-cancer cells lines (doxorubicin was used as a positive control) (**b**), effect of enzalutamide on MomC cytotoxicity in LNCaP cells (**c**, Chou–Talalay method), and colony formation assay (**d**).

Recently, a new member of the pentacyclic guanidine alkaloid group, monanchoxymycalin C (MomC) (Fig. 1a), has been isolated from the marine sponge *Monanchora pulchra* by our group^21^. The compound was found to be cytotoxic to human cervical carcinoma HeLa cells at low micromolar concentrations, induced S-phase cell cycle arrest and was synergistic in combination with cisplatin^21^. In the current study, we evaluated MomC in human prostate cancer cell lines revealing different levels of drug resistance. We used a functional kinomics screening followed by validation experiments to explore the molecular targets and the mechanism of action of this alkaloid.

## Materials and methods

### Reagents and antibodies

Anisomycin was purchased from NeoCorp (Weilheim, Germany); z-VAD(OMe)-fmk from Enzo Life Sciences (Farmingdale, NY, USA); annexin-V-FITC from BD Bioscience (San Jose, CA, USA); MTT (3-(4,5-dimethylthiazol-2-yl)-2,5-diphenyltetrazolium bromide), doxorubicin, CCCP (2-[2-(3-chlorophenyl)hydrazinylyidene]propanedinitrile), and propidium iodide (PI) from Sigma (Taufkirchen, Germany); cOmplete™ *EASY*packs protease inhibitors cocktail and PhosSTOP™ *EASY*packs from Roche (Mannheim, Germany); androgen receptor antagonist enzalutamide from Hycultec GmbH (Beutelsbach, Germany). The MEK inhibitor PD98059 was purchased from Merck Chemicals GmbH (Darmstadt, Germany), ERK1/2 inhibitors FR180204 and SCH772984 from Adooq bioscience (Irvine, CA, USA); PARP inhibitor olaparib JNK1/2 inhibitor SP600125 and p38 inhibitor SB203580 from LC Laboratories (Woburn, MA, USA).

### Monanchoxymycalin C

Monanchoxymycalin C (MomC, Fig. 1a) was isolated from the marine sponge *Monanchora pulchra* (Lambe, 1894) collected during scientific expedition of the research vessel “Academic Oparin” (September 2016; Chirpoi Island; 46° 23, 8 N; 150° 47, 8 E) as reported previously. The structure of MomC was determinated on the basis of spectroscopic data^21^. MomC was identified by comparison of its ^1^H and ^13^C NMR data with the previously published^21^. The purity of MomC was verified by HPLC, ^1^H and ^13^C NMR spectroscopy. For the experiments, a sterile solution of MomC in 100% DMSO was used.

### Cell lines and culture conditions

Human prostate cancer cell lines PC-3, DU145, LNCaP, 22Rv1, and VCaP, human prostate non-cancer cells RWPE-1 and PNT2, as well as human non-cancer fibroblasts cells MRC-9 were purchased from ATCC (Manassas, VA, USA). For the experiments the cell passage ≤ 50 was used. PC-3, DU145, LNCaP, 22Rv1, and PNT2 cells were cultured in 10% FBS/RPMI medium (RPMI medium supplemented with Glutamax™-I (gibco^®^ Life technologies™, Paisley, UK), 10% FBS (gibco^®^ Life technologies™) and 1% penicillin/streptomycin (gibco^®^ Life technologies™). VCaP and MRC-9 cells were cultured in 10% FBS/DMEM medium (RPMI medium supplemented with Glutamax™-I (gibco^®^ Life technologies™, Paisley, UK), 10% FBS and 1% penicillin/streptomycin). RWPE-1 cells were cultured in Keratinocyte Serum Free Medium (K-SFM) kit (gibco^®^ Life technologies™, Paisley, UK, Cat. #17005-042) supplemented with BPE and hEGF and 1% penicillin/streptomycin). The cell lines were recently authenticated by Multiplexion GmbH (Heidelberg, Germany). In all the experiment the control cells were pre-treated/treated with an equal amount of vehicle (DMSO).

### Flow cytometry analysis

The experiment was performed as previously reported^22^. 22Rv1 cells were seeded in 6-well plates (0.2 × 10^6^ cells/well in 2 mL/well). Following overnight incubation the cells were pre-treated with 100 µM z-VAD(OMe)-fmk for 1 h in 2 mL/well of fresh media. Censequantly, the cells were treated with the investigated drugs for 48 h and harvested by trypsinisation. Cells were immediately stained with annexin-V-FITC and propidium iodide and further analyzed using FACS Calibur instrument (BD Bioscience, San Jose, CA, USA). The results were quantified using the Cell Quest Pro software v. 5.2.1. (BD Bioscience).

### MTT assay

The experiment was performed as reported before^23^. 6,000 cells/well were seeded in a 96-well plate. Cells were incubated overnight and treated with MomC or vehicle (DMSO) in 100 μL/well of corresponding fresh culture media. Cells were consequently incubated with MTT solution (3-(-4,5-dimethylthiazol-2-yl)-2,5-diphenyltetrazolium bromide) for 2–4 h. The culture media was removed and the plates were dried for 1 h. To dissolve the formazan crystals the 50 µL/well of 100% DMSO were added and the optical density was measured using a spectrophotometer Infinite F200PRO reader (TECAN, Männedorf, Switzerland).

### Light microscopy

Cells were seeded in the 96-well plate and treated with the investigated drugs or vehicle for 48 h as described for MTT assay (see above). Microphotographs of the alive cells were taken using Axiovert 25 (Carl Zeiss, Göttingen, Germany) microscope, AxioCam MRc camera (Carl Zeiss) and AxioVision software v. 4.8.2 SP3 (Carl Zeiss) at 100 × magnification as previously reported^24^. The original images were croped and the figures were further prepared using the CorelDRAW X7 software v. 17.1.0.572 (Corel Corporation, Ottawa, Canada).

### Colony formation assay

Colony formation assay was performed as previously reported^24^. 22Rv1 or PC-3 cells were seeded in ø 6 cm TC Petri dishes (Sarstedt, Numbrecht, Germany; 1 × 10^6^ cells/dish in 5 mL/dish) and treated with indicated concentration of MomC for 48 h. Then, 100 alive cells were seeded in 6-well plates im 2 mL/well and incubated for 14 days. Surviving colonies were fixed with 100% MeOH, stained with Giemsa solution and counted by naked eye.

### Western blotting

The experiment was performed as previously described^25^. In brief, 1 × 10^6^ cells/dish cells were seeded in ø 6 cm Petri dishes in 5 mL/dish of media. Cells were incubated overnight followed by treatment with indicated concentrations of MomC or anisomycin for 48 h. Cells were harvested by scraping, washed (2 × ice cold PBS), and lysed. The samples were shortly frozen and centrifuged at 10,000*g*. The protein concentrations in the supernatants were determined by Bradford assay. The samples (20–30 μg/lane) were separated using SDS-PAGE method in gradient Mini-PROTEAN^®^ TGX Stain-FreeTM gels (Bio-Rad, Hercules, CA, USA) at 200 V. The proteins were transferred to a PVDF membrane, the membranes were blocked and consequantly incubated with the primary and secondary antibodies. The signals were detected using the ECL chemiluminescence system (Thermo Scientific, Rockford, IL, USA). All procedures were performed according to the manufacturers’ protocols. β-Actin was used as a loading control. For the list of used antibody see Supplementary Table S1. The original images were croped and the figures were further prepared using the CorelDRAW X7 software v. 17.1.0.572 (Corel Corporation, Ottawa, Canada).

### Kinase activity profiling

Kinase activity profiling was performed as described before^26^. For profiling serine-/threonine kinases, STK-PamChip^®^ arrays and a PamStation^®^12 (PamGene International, ‘s-Hertogenbosch, The Netherlands) were used according to the manufacturer’s protocols. In brief, each array contains 140 individual peptide phospho-sites that are analogues of substrates for the corresponding serine-/threonine kinases. The whole cell lysates were prepared using M-PER Mammalian Extraction Buffer (Pierce, Waltham, Massachusetts, USA) containing Halt Phosphatase Inhibitor (Pierce) and EDTA-free Halt Protease Inhibitor Cocktail (Pierce). 1 µg of protein and 400 µM ATP were mixed and applied per each array. Sequence-specific peptide phosphorylation was detected using anti-phospho-Ser/Thr antibodies during the reaction followed by detection with secondary polyclonal swine anti-rabbit Immunoglobulin-FITC antibody (PamGene International). For the signal record a CCD camera and the Evolve software v. 1.0 (PamGene International). The quality of signals were controlled. For further data analysis the final signal intensities were log2-transformed and further proceeded using the BioNavigator software v. 6.0 (BN6, PamGene International).

### Determination of drug combination effects

The effects (synergistic, antagonistic, or additive) of MAPK inhibitiors (PD98059, SCH772984, SP600125, SB203580), PARP inhibitior olaparib, androgen receptor antagonist enzalutamide, or antioxidant *N*-acetyl-l-cysteine (NaC) on the cytotoxic activity of MomC was deterimed using the Chou-Talalay method^27,28^. The experiments were performed as described before^9,25^. For combinational experiment with enzalutamide the LNCaP cells were co-treated with different concentrations of MomC and enzalutamide in 100 µL/well for 48 h. For all the other experiments the 22Rv cells were pre-treated with the different concentrations of inhibitors or vehicle for 1 h in 50 µL/well, then 50 µL/well of MomC solution in media were added and the plates were incubated for another 48 h. 0.1% FBS/RPMI media was used to evaluate an effect on NaC on MomC cytotoxicity; for all the other inhibitors regular 10% FBS/RPMI media was used. The cytotoxicity was measured by MTT assay and then proceeded with CompuSyn software v. 1.0 (ComboSyn Inc., Paramus, NJ, USA). The calculated combinational index (CI) > 1.1 indicates an antagonistic effect of the inhibitor on MomC cytotoxicity (red dots); CI = 0.9 ~ 1.1 refers to additive effect (clear dots); CI < 0.9 suggests synergism (green dots). Fraction affected (Fa) and CI values are shown in Supplementary Tables S2–S7.

### Cell fractionation

The isolation of the cytosolic proteins was performed using the Cell Fractionation Kit (Cat. No ab109719, abcam, Cambridge, MA, USA) as reported before^22^. In brief, 22Rv1 cells were treated for 48 h and harvested by scratching. The further procedures were performed following the previously described protocol^29^. Generated cytosolic fraction was concentrated using Amicon^®^ Ultra-2 Centrifugal Filter device (Cat. No. UFC203024, Merck, Darmstadt, Germany). Cells treated with 50 µM of CCCP for 48 h were used as a positive control.

### Determination of intracellular ROS levels

The levels of intracellular ROS were evaluated using a cell-permeable CM-H_2_DCFDA reagent (Molecular probes, Invitrogen, Eugene, OR, USA). The experiment was performed as previously reported^22,25^. In brief, the cells were plated in 12-well plates (0.2 × 10^6^ cells/well; 1 mL/well of media). The cells were incubated overnight and pretreated with CM-H_2_DCFDA (4 µM; 0.5 mL/well) for 30 min at 37 °C in the dark. Then, the cells were treated with the indicated concentrations of MomC or H_2_O_2_ (200 µM) for 6 h in pre-warmed PBS (1 mL/well). Afterwards the cells were trypsinized and immediately analyzed using FACS Calibur instrument and the Cell Quest Pro v.5.2.1. software.

### Statistical analysis

The data was analyzed using GraphPad Prism software v. 7.05 (GraphPad Prism software Inc., La Jolla, CA, USA). The values are presented as mean ± SEM. One-way ANOVA test followed by Sidak’s multiple comparison test was used for group comparison. Differences were considered to be statistically significant: *p < 0.05; **p < 0.01; ***p < 0.001. All experiments were performed in triplicates.

## Results

### MomC is cytotoxic to human drug-resistant prostate cancer cells

The cytotoxic activity of MomC (Fig. 1a) was investigated in the five prostate cancer cell lines PC-3, DU45, 22Rv1, VCaP, and LNCaP, possessing varying resistance profiles to standard therapies, as well as prostate non-cancer cell lines RWPE-1 and PNT2, and non-cancer human fibroblasts MRC-9 (Fig. 1b). Cytotoxicity was found in all cells tested at micro- or submicromolar concentrations following 48 h of treatment (Fig. 1b). At the same time, no significant selectivity towards cancer cells was observed in the in vitro experiments (Fig. 1b). PC-3 and DU145 cells are known to be unresponsive to androgen receptor (AR)-targeting therapy due to a lack of AR expression^30^, while AR splice variant seven mediates resistance of 22Rv1 and VCaP cells due to a loss of the androgen binding site^30^. Of note, AR-V7 induces an activation of the AR pathway independently of androgen binding^31^. Thus, AR targeting drugs such as abiraterone and enzalutamide are mostly ineffective^31^. LNCaP is a hormone dependent cell line harboring the natural expression of the full length AR (AR-FL)^30^. Interestingly, the cytotoxic activity of MomC correlated with the AR expression as follows: LNCaP (AR-FL^**+**^, AR-V7^**−**^) > 22Rv1 and VCaP (AR-V7^**+**^, AR-FL^**+**^) > PC-3 and DU145 (AR-FL^**−**^, AR-V7^**−**^) (Fig. 1b). Of note, the cytotoxic activity of MomC correlated with the AR status of the different cell line. Thus, highest sensitivity was observed in hormone sensitive LNCAP cells (AR-FL^**+**^/AR-V7^**−**^) while hormone-independent (i.e. AR-FL^**−**^ (PC3, DU145) or AR-V7^**+**^ (VCAP, 22Rv1) were slightly more resistant. Moreover, in line with this we have shown that the inhibition of AR activity in hormone-dependent LNCaP cells by treatment with enzalutamide (AR antagonist) could antagonize the MomC cytotoxic activity (Fig. 1c). Therefore, we speculate that in prostate cancer cells MomC cytotoxicity correlates with AR activity.

Colony formation of human prostate cancer cells was examined as a model for potential anti-metastatic activity of MomC. At non-cytotoxic concentrations the compound reduced the colony formation of 22Rv1 and PC-3 cells. Remarkably, active concentrations for PC-3 cells were 2- to 50-fold less than the cytotoxic IC_50_ for the corresponding cell lines (Fig. 1d).

### MomC induces non-apoptotic cell death of PCa cells

**Figure 2:**
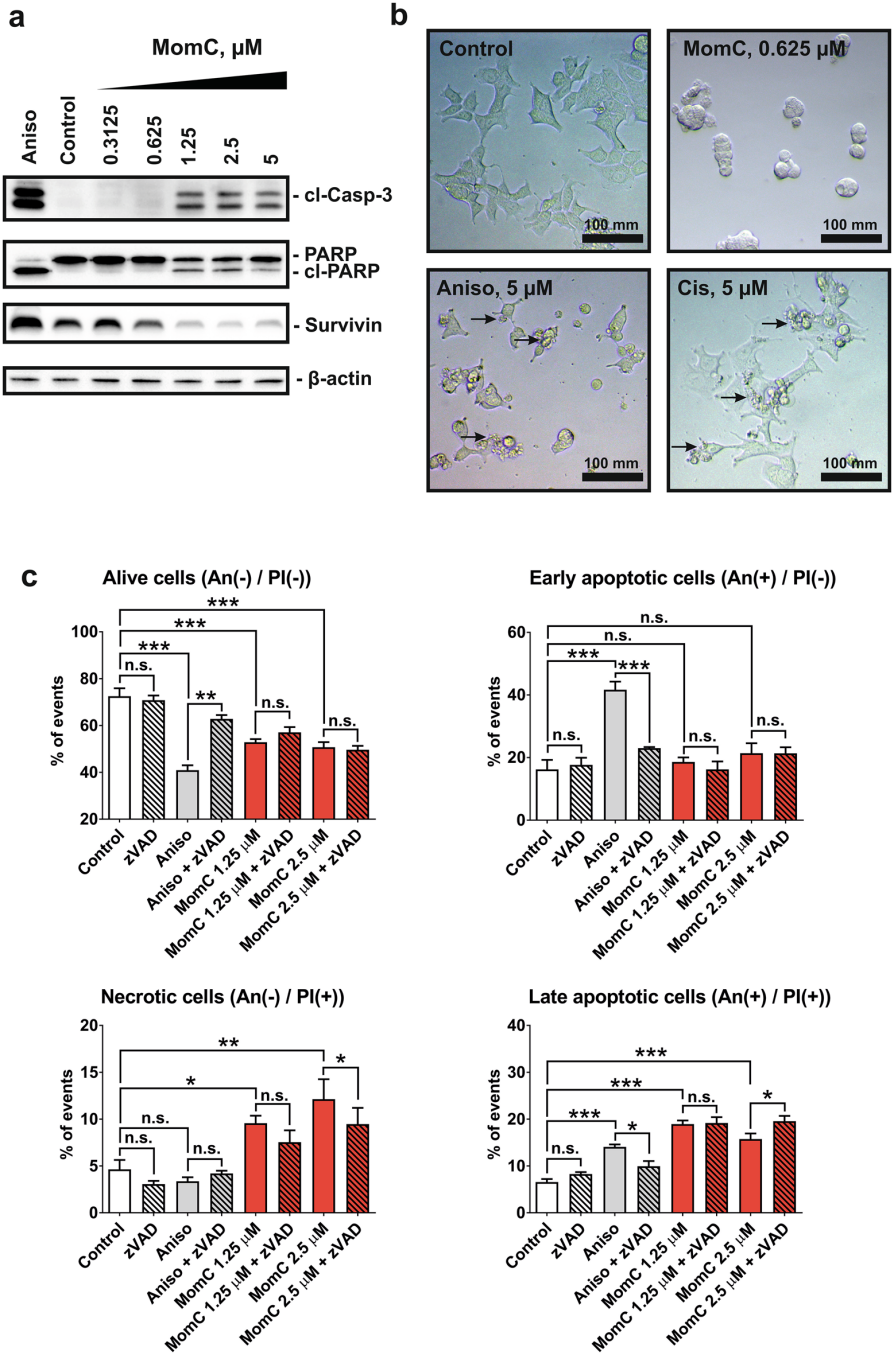
Western blotting analysis of apoptosis-related proteins (**a**) and morphological changes (**b**) in 22Rv1 cells treated with MomC, anisomycin (Aniso), or cisplatin (Cis). Analysis of 22Rv1 cells treated with MomC ± z-VAD(OMe)-fmk (zVAD) using flow cytometry analysis and annexin-V-FITC/PI double staining (**c**). The full-length blots are presented in Supplementary Fig. 1S.

The mechanisms of MomC-induced cell death were further investigated in drug-resistant 22Rv1 cells. 22Rv1 cells are known to be androgen-independent due to the expression of AR-V7, and therefore resistant to hormonal therapy^30^. Two apoptotic hallmarks, PARP cleavage and cleaved caspase-3, were detected after 48 h of MomC treatment (Fig. 2a), whereas anti-apoptotic protein survivin was downregulated (Fig. 2a). However, the morphology of the cells treated with MomC was markedly different from those treated with anisomycin (a well-established inducer of “classical” caspase-dependent apoptosis^32,33^ or cisplatin. Thus, regular apoptotic features such as cell shrinking, membrane blebbing, and apoptotic bodies formation were not detected in MomC-treated cells (Fig. 2b); in contrast, cell rounding and swelling was observed under the treatment (Fig. 2b).

Furthermore, an increased annexin-V-FITC^+^/PI^+^ and annexin-V-FITC^**−**^/PI^+^ cell populations under the MomC treatment were observed by flow cytometry indicating fraction of late apoptotic and necrotic cells. Note, PI^+^ staining indicates a loss of the cellular membrane integrity, being a feature of non-apoptotic cell death. At the same time, no significant increase of the Annexin-V-FITC^+^/PI^**−**^ cell population (early apoptosis) was detected in the MomC-treated samples, while in contrast this cell fraction was observed secondary to anisomycin treatment (positive control) as expected (Fig. 2c). In order to determine the role of caspases in this process, a co-treatment with well-established pan-caspase inhibitor z-VAD(OMe)-fmk was applied. Interestingly, the inhibitor did not influence the cytotoxic effects of MomC, while the activity of anisomycin was significantly reduced. Expectedly, z-VAD(OMe)-fmk pretreatment resulted in an increase of the alive cell population and a decrease of the early and late apoptotic cell population of anisomycin treated cells (Fig. 2c). These results indicate that MomC-induced cell death was exerted via non-apoptotic mechanisms and independently from caspase activities.

### MomC-induced alterations of protein tyrosine kinases activity

**Figure 3:**
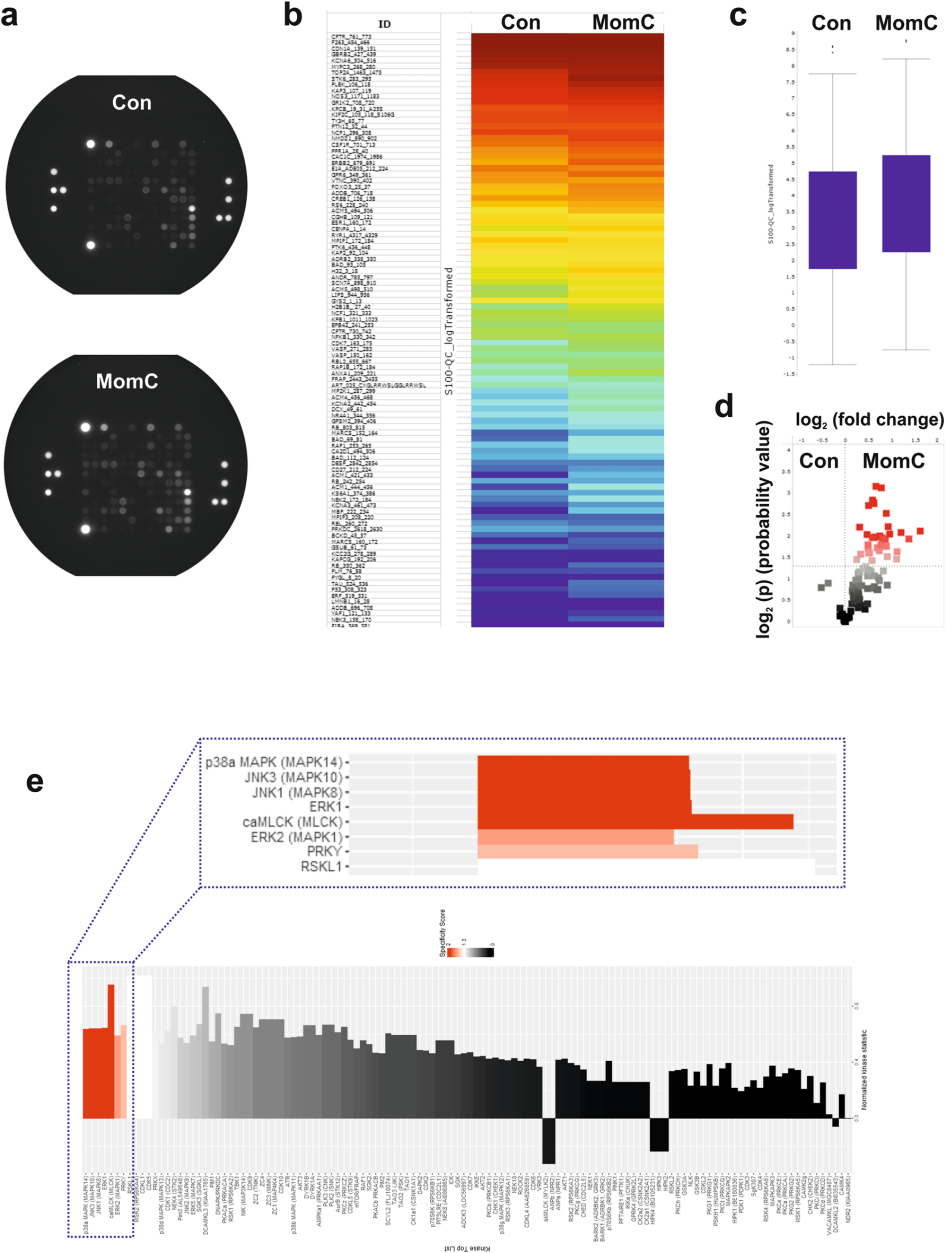
Functional kinome profiling of serine/threonine kinases (STK) in 22Rv1 cell treated with MomC for 2 h. Proteins were extracted and analyzed using STK-PamChip^®^ (sequence-specific peptide phosphorylation assay) and anti-phospho-STK antibodies (**a**). Heat-map plot (**b**), box plot (**c**), or volcano-plot (**d**) were constructed. Red squares indicate substrate peptides which have significantly higher phosphorylation (log_2_(p) > 1.3, dotted line) compared to control (**d**). Upstream analysis of kinases affected under the treatment (**e**). Normalized kinase statistic > 0: higher kinase activity in MomC-treated cells; specificity score > 1.3 (white to red bars): statistically significant changes (**e**).

Protein kinases catalyze the phosphorylation of specific proteins thereby modulating their activity. They play a critical role in numerous cancer-related events^34^. Unsurprisingly, they have become targets for anticancer therapeutics with many of them being clinically approved to date^34^. Since serine-/threonine kinases (STK) play a critical role in processes of cell death and survival, we systematically analyzed the STK activity performing functional kinomics^35^. We used the PamTechnology^®^ (https://www.pamgene.de, Fig. 3a) which allows the identification of kinases specifically activated or inhibited by the drug. For the experiment, a short drug exposure time of 2 h was chosen, to ensure, that only specific primary effects of MomC on the kinome of prostate cancer cells were identified, while the number of measured secondary effects resulting from cell death related processes was minimized. The results are presented as a log2 of signal intensity per peptide for each treatment group in Fig. 3b and summarized in Fig. 3c. The data indicate overall increased STK activity in MomC-treated samples compared to the control group (Fig. 3c, 3d). Note, no significant treatment-induced inhibition of phosphorylation was observed for any peptide tested (Fig. 3d).

Analyses of upstream kinases potentially influenced by MomC predicted the activation of several kinases belonging to the family of mitogen-activated protein kinase (MAPK), namely p38, JNK1/3, and ERK1/2—all known to be involved in different cancer-related processes^34,36^. In addition, a cardiac myosin light chain kinase (caMLCK), RSK-like pseudokinase 1 (RSKL1), and Y-linked protein kinase (PRKY) were predicted to be activated (Fig. 3e).

caMLCK is a kinase which is important for muscle contraction (in particular heart)^37^. There is few information on its role in cancer. Thus, a related kinase—i.e. non-muscular myosin light chain kinase (nmMLCK) was reported to be overexpressed in prostate cancer^38^. However, its role in cancer development and treatment seems to be controversial^39^. RSKL1 is a RSK-like pseudokinase 1, also known as ribosomal protein S6 kinase C1 (RPS6KC1). It may be involved in transmitting sphingosine-1 phosphate (SPP), which is an important cellular messenger^40^. Additionally, it can bind to peroxiredoxin-3 and may help to transport it to mitochondria and early endosomes^41^. However, no relation of this kinase with cancer progression or treatment prognosis was reported, even though the mutations of the correspondent gene were detected in several human cancers^42^. The activation of PRKY was predicted as well. The close analogue of this kinase, PRKX plays an important role in differentiation, epithelial morphogenesis, and is also involved in angiogenesis^43^. However, to the best of our knowledge, there is no known cellular function of PRKY as well as any reports on its expression in cancer cells.

### Validation of kinome analysis data

**Figure 4:**
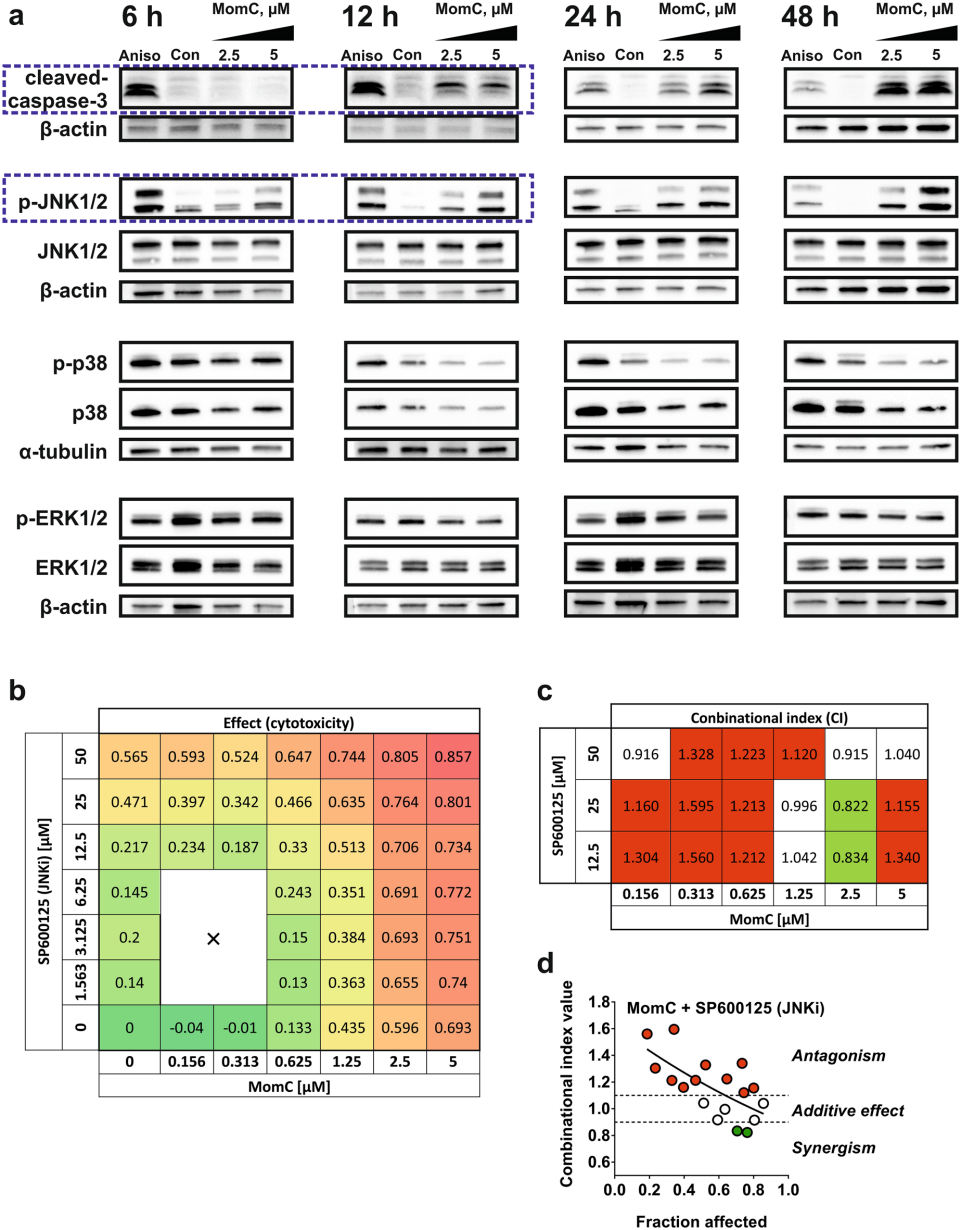
Validation of kinome analysis data was performed using Western blotting (**a**). The full-length blots are presented in Supplementary Fig. 2S. Cells treated with 10 µM anisomycin (Aniso) were used as a positive control. Cytotoxic effects of MomC and JNK1/2 inhibitor SP600125 alone and in combination (effect = 0 corresponds to 100% cell viability; effect = 1 corresponds to 0% cell viability) (**b**). Combinational index (CI) values calculated using Chou–Talalay method (**c**, **d**).

All top-5 ranked kinases belong to the family of mitogen-activated protein kinases (Fig. 3e). Thus, p38, JNKs, and ERKs were predicted to be predominantly activated by MomC in cancer cells (Fig. 3e). In order to validate these findings, Western blotting analyses were performed for different MomC treatment periods (up to 48 h) (Fig. 4a). Indeed, a dose- and time-dependent activation of JNK1/2 was observed starting as early as 6 h after treatment initiation. In contrast, no caspase-3 activation was found at that time. Of note, no alterations of ERK1/2 or p38 activation were detected—even after 48 h of treatment. An inhibition of both phosphorylated and total p38 was attributed to cytotoxic processes, starting to be pronounced 12 h after treatment initiation (Fig. 4a).

Depending on the nature of the stimulus and the model used an activation of JNK1/2 and other MAPKs may exert either pro-apoptotic or pro-survival function^44^. In order to determine the specific effects in the prostate cancer cells, we performed co-treatment of the cells with an established JNK1/2 inhibitor SP600125. Due to the weak but still detectable cytotoxicity of SP600125 alone in 22Rv1 cells, a Chou-Talalay method was implemented. This method is often applied for the evaluation of the effects of two or more drug used in combination (i.e. synergistic, additive, or antagonistic effect)^28^. It uses an isobologram equation for calculation of combinational index (CI). Hence, we created a heat-map of cytotoxic activity of MomC and SP600125 alone and in combination (Fig. 4b). Further analysis of the data revealed an antagonistic effect of SP600125 on MomC-induced cytotoxicity in 22Rv1 cells at the whole range of concentration of the investigated marine alkaloid (Fig. 4c). Thus, an activation of JNK1/2 under the treatment was determined to be a pro-cytotoxic stimulus.

### Role of ROS and PARP in MomC-induced cells death

**Figure 5:**
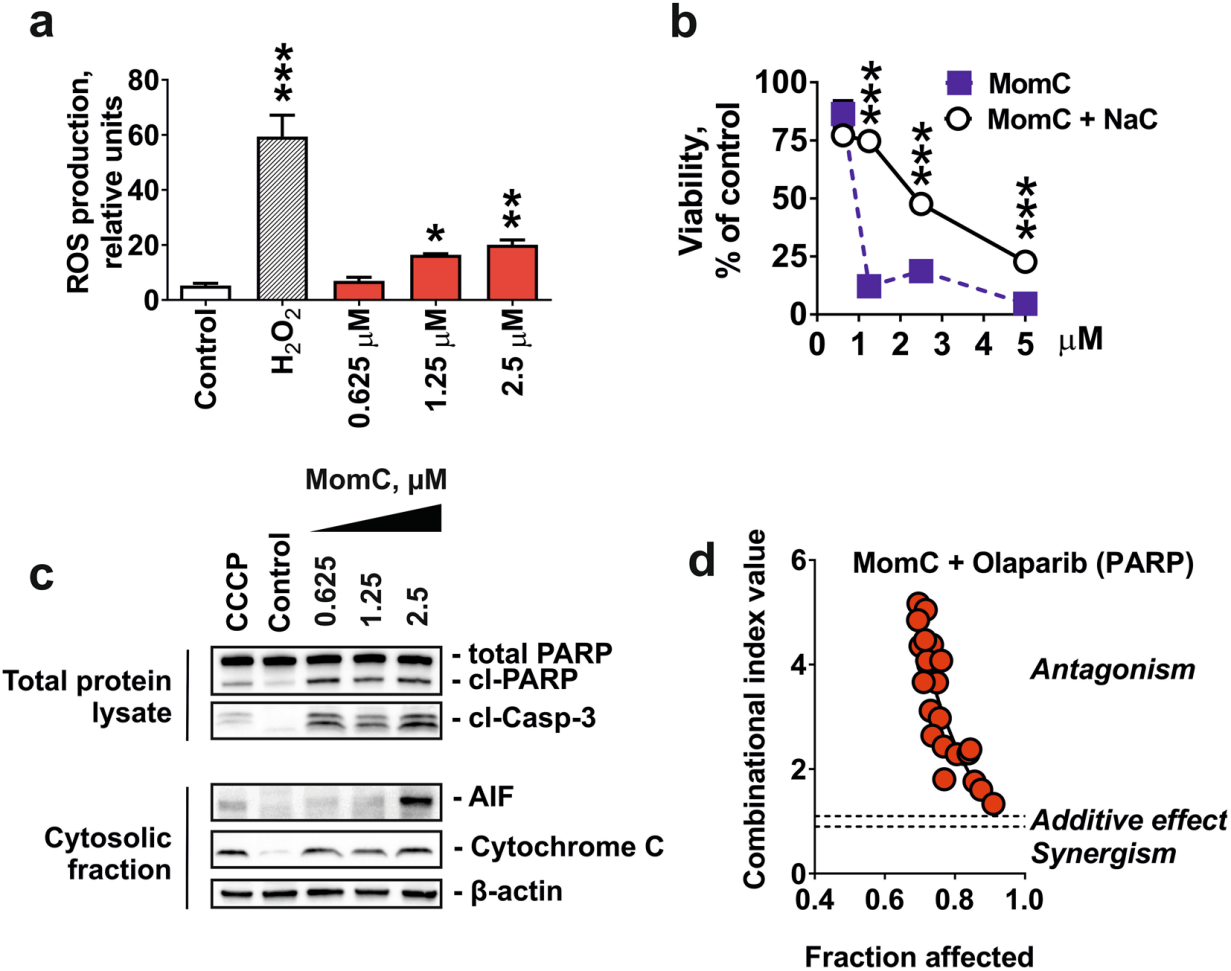
Effect of MomC on ROS production (**a**) and mitochondria integrity, indicated by release of the mitochondrial proteins to cytoplasm (**c**). The full-length blots are presented in Supplementary Fig. 3S. NaC (1 mM, MTT assay) (**b**) and olaparib (Chou–Talalay method) (**d**) could effectively antagonize the cytotoxic effect of MomC.

As JNKs activation may result from oxidative stress^45^ we examined ROS levels in MomC-treated 22Rv1 cells. Indeed, an upregulation of ROS was detected after 6 h of treatment (Fig. 5a). Moreover, pre-treatment of the cells with an established antioxidant *N*-acetyl-l-cysteine (NaC) significantly antagonized cytotoxic effects of MomC (Fig. 5b). Thus, ROS activation significantly contributes to the anticancer activity of the investigated marine alkaloid. In line with this, we detected a release of cytotoxic mitochondrial proteins to cellular cytoplasm, e.g. cytochrome C and apoptosis inducing factor (AIF), which was correlating with the induction of cell death hallmarks (caspase-3- and PARP-cleavage) (Fig. 5c). This indicates MomC-induced mitochondria membrane permeabilization, which could be either a reason or a result of the elevated ROS production^46^. An excessive intracellular ROS may induce ssDNA break, which could, however, further be repaired by PARP, thereby rescuing a cancer cell from DNA damage-induced apoptosis^47^. Therefore, we examined the effect of PARP inhibitor olaparib on MomC activity. However, the expected synergistic effect of olaparib was not observed, but on the contrary, a pronounced antagonistic effect was detected (Fig. 5d). Of note, a similar effect was previously reported for ROS-induced non-apoptotic cell death, executed via consecutive induction of JNKs and sustained PARP1 activation^45^.

### Role of other MAPK in MomC-induced cytotoxicity

**Figure 6:**
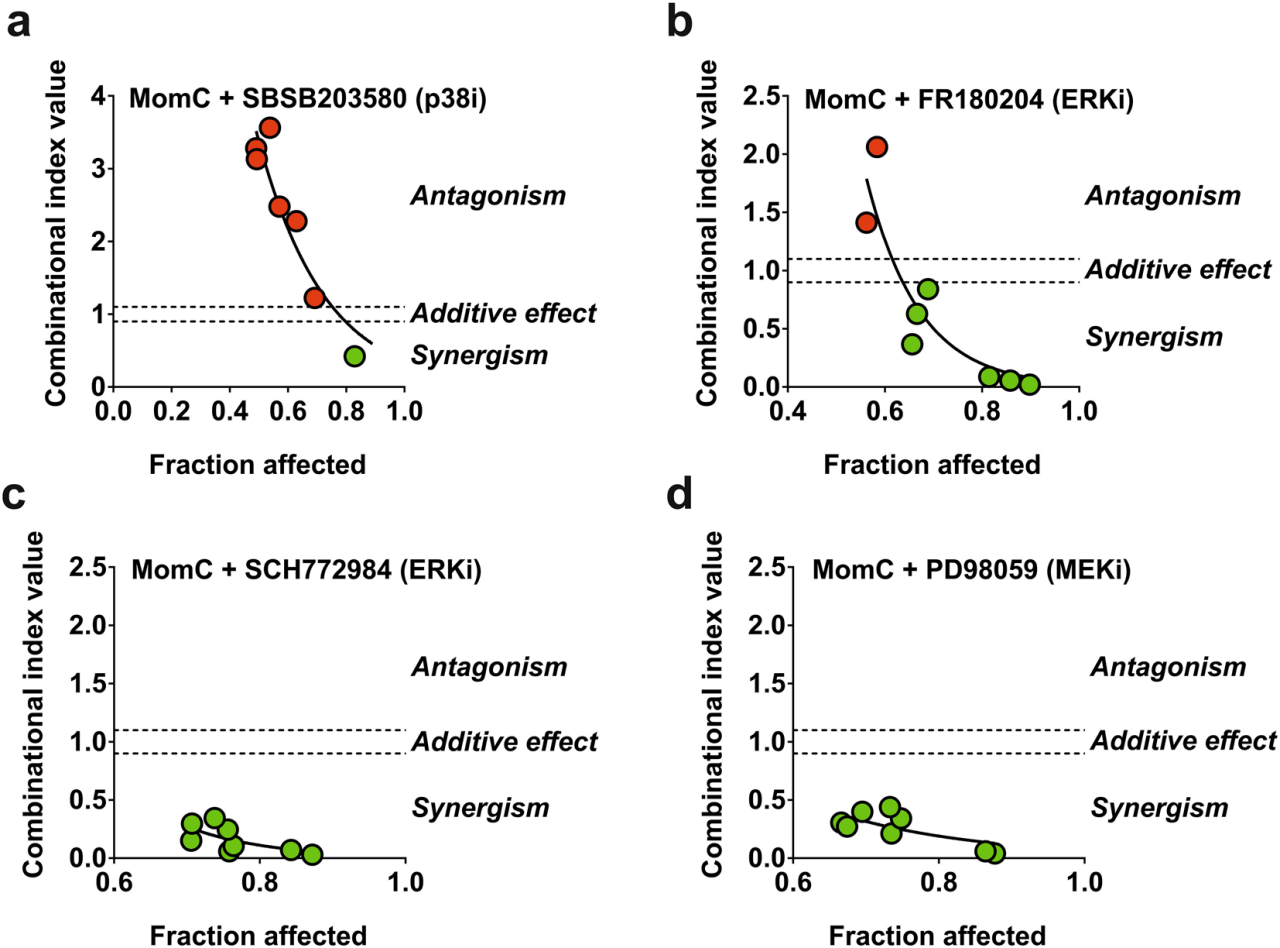
Analysis of the effect of ERK1/2 inhibitors FR180204 (**a**) and SCH772984 (**b**), MEK1/2 inhibitor PD98059 (**c**), or p38 inhibitor SB203580 (**d**) on MomC activity (Chou–Talalay method).

Next, we evaluated the impact of the pathways involving other MAPKs, predicted to be activated by kinome analysis (Fig. 3e), but without significant changes in the validation Western blotting analyses (Fig. 4a). Therefore, we applied p38 inhibitor SP203580 as well as ERK1/2 inhibitors FR180204 and SCH772984 in combination with MomC. Additionally, MEK1 inhibitor PD98039 was used. MEK1/2 kinase is known to directly and exclusively phosphorylate ERK1/2^48^. Thus, inhibition of MEK1/2 ultimately results in ERK1/2 inactivation^48^, and PD98039 is often used as an indirect ERK1/2 inhibitor. Due to the slight cytotoxic effects of these inhibitors alone, the Chou-Talalay method was used. We observed an antagonistic effect of SP203580 (p38i) on MomC activity in 22Rv1 cells (Fig. 6a). On the other hand, we detected synergistic effects of FR180204 (ERKi, Fig. 6b), SCH772984 (ERKi, Fig. 6c), as well as of PD98039 (MEK/ERKi, Fig. 6d) on the activity of the investigated alkaloid. This suggests that p38 kinase is involved in the cellular processes which are important for the MomC-induced cytotoxicity, whereas ERK1/2 mediates rather pro-survival processes (Fig. 6a–d).

## Discussion

In the current study, we demonstrate cytotoxicity of recently isolated marine alkaloid MomC in PCa cells revealing different levels of resistance to approved standard therapies including taxanes and AR-targeting drugs such as abiraterone and enzalutamide. Effects were mediated by caspase-independent cytotoxic processes and exerted via activation of JNK1/2, a member of the mitogen-activated protein kinases (MAPKs).

Protein kinases modify target proteins by phosphorylation resulting in changes of enzyme activity, cellular location and interaction with other proteins. Due to this key function they play an important role in cancer development and growth^49^. Among them, MAPKs are a prominent family well known to be involved in various cancer-related biological processes as well as development of drug resistance and depletion of oncogene-induced senescence^34^. For instance, c-Jun N-terminal protein kinases (JNKs)—a subset of MAPK—affect cell survival and death, proliferation, migration, differentiation and inflammation processes by interaction with different nuclear and non-nuclear proteins^50^. Typical JNKs’ targets include c-Myc, p53, 14-3-3, Elk1, histone H3, histone H2AX, Bcl-2 family members, as well as several components of AP-1 (activator protein-1; a transcription factor) such as c-Jun and ATF2 (reviewed in Ref.^50^). In cancer, JNKs have been described to be a “double-edged sword”. Thus, in addition to a well-established and most often observed pro-apoptotic activity, anti-apoptotic functions were reported as well^44^. The effect of JNKs on cell viability seems to be dependent on the death stimulus’ nature, cell type, duration of activation, and the status of the other signaling pathways^44^.

In prostate cancer, crucial effects on tumor growth have been assigned to specific members of the MAPK family^36,51,52^ and tight interactions with AR-signaling have been demonstrated^53,54^—in particular, a crosstalk between AR and JNK^55^. Thus, activation of the AR pathway led to the suppression of JNK activation followed by inhibition of apoptosis in PCa cells^55^. In contrast, an active JNK was required to maintain the sensitivity of human PCa cells to androgens and androgen-receptor targeting drugs^56^. Moreover, anti-apoptotic activity of androgens in PCa were at least in part exerted via inhibition of JNKs^57^. Hence, JNK suppression contributes to androgen-mediated survival of PCa cells^57^. In addition, JNK inactivation or deletion promoted the development of aggressive androgen-independent metastatic prostate cancer in vivo. Thus, JNK inhibition seems to be an unfavorable event in prostate cancer therapy^58^. First clinical data confirm these findings. Thus, a higher intratumoral expression of active phospho-JNKs correlated with a survival advantage of prostate cancer patients^59^.

To date, both, intrinsic and extrinsic mediated apoptosis, as well as non-apoptotic/necrotic cell death have been reported following JNKs activation in PCa cells. While Bcl-2 phosphorylation and degradation, mitochondrial cytochrome C release and caspase-9 activation are involved in the intrinsic pathway^60^, extrinsic apoptosis is exerted by modulation of the TNFα- or FasL-Fas-mediated pathways^51,61^. In addition, endoplasmic reticulum (ER) targeting was described to result in JNKs activation resulting in apoptosis of prostate cancer cells (via Ca^2+^ release induced JNK activation followed by the cleavage of executioner caspases; or ER stress induced IRE-1/ASK1/JNK signaling followed by intrinsic apoptosis)^51^. Non-apoptotic cell death was reported to be initiated under excessive ROS and executed via JNK-mediated PARP1 activation which ultimately lead to the cell death^45^.

In prostate cancer, approximately ten JNK-activating natural compounds (e.g. jungermannenone B^62^, capilliposide C^63^, guttiferone F^64^ and others^51^) have been identified to date and were reported to induce cell cycle arrest and apoptosis in vitro. Of note, for some of these substances, i.e. curcumin and costunolide, synergism with standard chemotherapeutics used in the treatment of prostate cancer was observed (reviewed in Ref.^51^). In our study, the kinome screening identified MAPKs as potential direct or indirect targets of the marine natural compound MomC in PCa cells. Indeed, validation experiments revealed an activation of JNK1/2, which was relevant for MomC-induced cytotoxicity. Remarkably, this activation was observed shortly after treatment initiation and prior to cell death related events. Moreover, inhibition of JNKs activity by SP600125 effectively antagonized MomC cytotoxicity. Hence, JNK1/2 activation is not the result of the other cell death related events (e.g. caspases activation), but rather its initiator.

Remarkably, we previously reported the cytotoxic activity of MomC in human cervical carcinoma HeLa cells^21^. In that study an apoptosis-like character of the MomC-induced cell death was suggested^21^. Interestingly, in both, cervical^21^ and prostate cancer cells (Fig. 2a–c) apoptotic hallmarks such as cleavage of caspase-3 and PARP were observed. However, more precise and detailed examinations suggested cancer cell elimination rather by non-apoptotic mechanisms, at least in PCa: this non-apoptotic character was indicated by (i) distinct morphology of the treated cells (cell rounding and swelling, lack of shrinking and blebbing, lack of apoptotic bodies formation), (ii) independence of the cytotoxic effect from caspase activity, (iii) increase of late apoptotic and necrotic cell fraction under MomC treatment, while no early apoptosis was detected. In fact, the detected caspase-3- and PARP-cleavage were most likely unspecific events secondary to other cell death related processes (e.g. mitochondria or lysosomal membrane permeabilization). Of note, the mechanism of the induced cytotoxic effects may be cell-specific and therefore distinct in the different models. The mode of MomC action in the human cervical carcinoma HeLa cells therefore awaits further examination and clarification.

Notably, we observed an upregulation of cytotoxic ROS and mitochondrial damage under drug exposure. In addition, active PARP was required for MomC-induced cytotoxic activity. Thus, the increased ROS production did not cause the classical apoptotic cell death mediated by the induction of ssDNA breaks, but rather a specific JNKs activation resulting in further PARP activation. Indeed, previous reports demonstrate that excessive ROS production leads to JNK activation followed by sustained PARP activation^45^. Consequences are a quick depletion of cellular NAD^+^ resulting in ATP production failure and cell death of a non-apoptotic character^65^. Indeed, our findings indicate that MomC exerts its activity this way.

Interestingly, the cytotoxic activity of MomC in PCa correlated with the AR status of the cell lines, suggesting a functional AR pathway to contribute to the execution of the cytotoxic program. This can at least be partly explained by a cross-talk between AR and JNK. Indeed, JNK activation was reported to be downregulated with an increasing grade of PCa^57^. In addition, antiapoptotic activity of androgens in both androgen-dependent and -independent PCs was found to be executed via downstream blocking of JNK activation^55,57^.

p38 and ERK1/2 are additional MAPK involved in different cellular processes, which mediate either pro-cytotoxic or pro-survival processes depending on the stimuli and cellular context^36,49,66^. In prostate cancer, the activation of p38 by chemotherapy or other stress stimuli negatively regulates cell cycle progression, inhibits migration and promotes apoptosis (reviewed in Ref.^36^). In contrast, in non-stress conditions p38 is critical for hypoxia-reoxygenation induced androgen-independent activation of AR, therefore contributing to the hormone resistance of PCa^67^. Moreover, an active p38 kinase contributes to survival, clonogenicity, migratory and invasive properties of PCa cells^36^. The role of ERK1/2 kinase in prostate cancer is described to be more univocal. Thus, it’s basal activity as well as activation are associated with advanced malignancy, initiation of prostate cancer development and cancer cell invasion^36^. Moreover, ERK1/2 activation is associated with promotion of AR-signaling and increased PSA expression^36^. In patients, active ERK1/2 was found to be associated with advanced disease with the highest levels reported for metastatic castration-resistant PCa (mCRPC). First signs of clinical activity were reported for MEK/ERK inhibition and a phase II clinical trial examining the activity of trametinib in mCRPC is currently recruiting (NCT02881242)^68^.

In our experiments, the kinome analysis predicted p38 and ERK1/2 to be also regulated by MomC; however, no significant changes of the phosphorylated forms were observed in validation experiments. Of note, a distinct level of active p-ERK1/2 was detected in non-treated (control) cells, with no obvious further regulation under treatment. However, cellular processes controlled by ERK1/2 even at its basal activity level may counteract the activity of MomC. Thus, several ERK inhibitors synergized with MomC increasing its cytotoxic effects. Therefore, MEK/ERK inhibitors can be considered for a combinational use with MomC in future drug development. In contrast, an inhibition of p38 activity by p38 inhibitor SP203580 antagonized cytotoxic activity of MomC. At the same time, p38 was not activated under the MomC treatment. Hence, p38 activity (even without additional activation) may be important for successful execution of the MomC-induced cytotoxic program. Finally, it should be noted that a possible activation of caMLCK, predicted by kinome analysis, may cause a stimulation of heart muscle contraction. This potential side effect needs to be carefully monitored in in vivo experiments and future clinical trials.

## Conclusions

Distinct from other natural compounds marine pentacyclic guanidine alkaloid MomC effectively kills PCa by induction of caspase-independent non-apoptotic cell death. The mitogen-activated protein kinase JNK1/2 was identified as one of the primary molecular targets with an early activation prior to other processes involved in MomC mediated cell death. Although no change of p38 and ERK1/2 activity was detected, p38 was shown to be important for cytotoxic activity of MomC, whereas inhibition of ERK1/2 increased cytotoxic effects of the marine compound. In conclusion, MomC is a promising novel JNK1/2 targeting marine compound for the treatment of advanced, drug resistant prostate cancer.

## Supplementary information

Supplementary information.
