# Supplementary material for: Marine alkaloid monanchoxymycalin C: a new specific activator of JNK1/2 kinase with anticancer properties
Source: Sci Rep. 2020 Aug 6;10:13178. doi: 10.1038/s41598-020-69751-z (PMC7411023; doi:10.1038/s41598-020-69751-z)
Supplement: Supplementary file 1 — Supplementary information. [file 41598_2020_69751_MOESM1_ESM.docx]

Supplementary information

Marine alkaloid Monanchoxymycalin C: a new specific activator of JNK1/2 kinase with anticancer properties

Sergey A. Dyshlovoy^1,2,3,4,^*, Moritz Kaune^1^, Malte Kriegs^5,6^, Jessica Hauschild^1^, Tobias Busenbender^1^, Larisa K. Shubina^2^, Tatyana N. Makarieva^2^, Konstantin Hoffer^5,6^, Carsten Bokemeyer^1^, Markus Graefen^4^, Valentin A. Stonik^2^, Gunhild von Amsberg^1,4^

^1^ Department of Oncology, Hematology and Bone Marrow Transplantation with Section Pneumology, Hubertus Wald Tumorzentrum – University Cancer Center Hamburg (UCCH), University Medical Center Hamburg-Eppendorf, Martinistrasse 52, 20246 Hamburg, Germany; s.dyshlovoy@uke.de (S.A.D.), moritz.kaune@stud.uke.uni-hamburg.de (M.Ka.), m.kriegs@uke.de (M.Kr.) j.hauschild@uke.de (J.H.), tobias.busenbender@gmx.de (T.B.), c.bokemeyer@uke.de (C.B.), g.von-amsberg@uke.de (G.v.A.)

^2^ G.B. Elyakov Paciﬁc Institute of Bioorganic Chemistry, Far-East Branch, Russian Academy of Sciences, 690022 Vladivostok, Russia; shubina@piboc.dvo.ru (S.L.K.), makarieva@piboc.dvo.ru (T.N.M.), stonik@piboc.dvo.ru (V.A.S.)

^3^ School of Natural Sciences, Far Eastern Federal University, 690091 Vladivostok, Russian Federation

^4^ Martini-Klinik, Prostate Cancer Center, University Hospital Hamburg-Eppendorf, Martinistrasse 52, 20246 Hamburg, Germany; graefen@martini-klinik.de (M.G.)

^5^ Department of Radiotherapy & Radiation Oncology, Hubertus Wald Tumorzentrum – University Cancer Center Hamburg (UCCH), University Medical Center Hamburg-Eppendorf, Martinistrasse 52, 20246 Hamburg, Germany; m.kriegs@uke.de (M.Kr.), k.hoffer@uke.de (K.H.)

^6^ UCCH Kinomics Core Facility, Hubertus Wald Tumorzentrum – University Cancer Center Hamburg (UCCH), University Medical Center Hamburg-Eppendorf, Martinistrasse 52, 20246 Hamburg, Germany

***** Correspondence to: Dr. Sergey A. Dyshlovoy, Laboratory of Experimental Oncology, Department of Oncology, Hematology and Bone Marrow Transplantation with Section Pneumology, Hubertus Wald Tumorzentrum – University Cancer Center Hamburg (UCCH), University Medical Center Hamburg-Eppendorf, Campus Forschung (N27, room 04.082), Martinistrasse 52, 20246 Hamburg, Germany; Tel.: +49-40-7410-51950; e-mail: or s.dyshlovoy@uke.de; dyshlovoy@gmail.com.

**Table S1.** The list of antibodies used.

| **Antibodies** | **Clonality** | **Source** | **Cat.-No.** | **Dilution** | **Manufacturer** |
| --- | --- | --- | --- | --- | --- |
| anti-AIF | mAb | rabbit | #5318 | 1:1000 | Cell Signaling |
| anti-cleaved Caspase-3 | mAb | rabbit | #9664 | 1:1000 | Cell Signaling |
| anti-cytochrome C | mAb | rabbit | #11940 | 1:1000 | Cell Signaling |
| anti-mouse IgG-HRP |  | sheep | NXA931 | 1:10000 | GE Healthcare |
| anti-PARP | pAb | rabbit | #9542 | 1:1000 | Cell Signaling |
| anti-rabbit IgG-HRP |  | goat | #7074 | 1:5000 | Cell Signaling |
| anti-Survivin | pAb | rabbit | NB500-201 | 1:1000 | Novus |
| anti-α-Tubulin | mAb | mouse | T5168 | 1:5000 | Sigma-Aldrich |
| anti-ERK1/2 | mAb | mouse | #9107 | 1:2000 | Cell Signaling |
| anti-JNK1/2 | mAb | rabbit | #9258 | 1:1000 | Cell Signaling |
| anti-p38 | mAb | rabbit | #9212 | 1:1000 | Cell Signaling |
| anti-phospho-ERK1/2 | mAb | rabbit | #4377 | 1:1000 | Cell Signaling |
| anti-phospho-JNK1/2 | mAb | rabbit | #4668 | 1:1000 | Cell Signaling |
| anti-phospho-p38 | mAb | rabbit | #4511 | 1:1000 | Cell Signaling |
| anti-α-Tubulin | mAb | mouse | T5168 | 1:5000 | Sigma-Aldrich |
| anti-β-Actin-HRP | pAb | goat | sc-1616 | 1:10000 | Santa Cruz |

**Table S2.** Fraction affected (Fa) and CI values calculated for combination of MomC + Olaparib

| **MomC [µM]** | **Olaparib [µM]** | **Fa** | **CI value** |
| --- | --- | --- | --- |
| 1.25 | 12.5 | 0.731 | 3.118 |
| 2.5 | 12.5 | 0.757 | 2.973 |
| 5 | 12.5 | 0.826 | 2.290 |
| 1.25 | 25 | 0.724 | 4.001 |
| 2.5 | 25 | 0.749 | 3.657 |
| 5 | 25 | 0.839 | 2.303 |
| 1.25 | 50 | 0.724 | 4.001 |
| 2.5 | 50 | 0.749 | 3.657 |
| 5 | 50 | 0.839 | 2.303 |
| 1.25 | 100 | 0.724 | 4.001 |
| 2.5 | 100 | 0.749 | 3.657 |
| 5 | 100 | 0.839 | 2.303 |
| 1.25 | 12.5 | 0.724 | 4.001 |
| 2.5 | 12.5 | 0.749 | 3.657 |
| 5 | 12.5 | 0.839 | 2.303 |
| 1.25 | 25 | 0.724 | 4.001 |
| 2.5 | 25 | 0.749 | 3.657 |
| 5 | 25 | 0.839 | 2.303 |
| 1.25 | 50 | 0.724 | 4.001 |
| 2.5 | 50 | 0.749 | 3.657 |
| 5 | 50 | 0.839 | 2.303 |
| 1.25 | 100 | 0.724 | 4.001 |
| 2.5 | 100 | 0.749 | 3.657 |
| 5 | 100 | 0.839 | 2.303 |

**Table S3.** Fraction affected (Fa) and CI values calculated for combination of MomC + FR180204

| **MomC [µM]** | **FR180204 [µM]** | **Fa** | **CI value** |
| --- | --- | --- | --- |
| 0.625 | 6.25 | 0.562 | 1.41159 |
| 1.25 | 12.5 | 0.584 | 2.06078 |
| 2.5 | 25.0 | 0.689 | 0.83997 |
| 5.0 | 50.0 | 0.858 | 0.05468 |
| 0.625 | 12.5 | 0.656 | 0.36643 |
| 1.25 | 25.0 | 0.666 | 0.62870 |
| 2.5 | 50.0 | 0.815 | 0.08726 |
| 5.0 | 100.0 | 0.898 | 0.01950 |

**Table S4.** Fraction affected (Fa) and CI values calculated for combination of MomC + PD98059

| **MomC [µM]** | **PD98059 [µM]** | **Fa** | **CI value** |
| --- | --- | --- | --- |
| 0.625 | 12.5 | 0.666 | 0.30586 |
| 1.25 | 25.0 | 0.735 | 0.21224 |
| 2.5 | 50.0 | 0.748 | 0.34148 |
| 5.0 | 100.0 | 0.877 | 0.04107 |
| 0.625 | 6.25 | 0.674 | 0.27234 |
| 1.25 | 12.5 | 0.695 | 0.39869 |
| 2.5 | 25.0 | 0.733 | 0.43866 |
| 5.0 | 50.0 | 0.864 | 0.05947 |

**Table S5.** Fraction affected (Fa) and CI values calculated for combination of MomC + SB203580

| **MomC [µM]** | **SB203580 [µM]** | **Fa** | **CI value** |
| --- | --- | --- | --- |
| 0.625 | 12.5 | 0.491 | 3.28048 |
| 1.25 | 25.0 | 0.571 | 2.47947 |
| 2.5 | 50.0 | 0.692 | 1.22253 |
| 5.0 | 100.0 | 0.889 | 0.48080 |
| 0.625 | 6.25 | 0.493 | 3.13114 |
| 1.25 | 12.5 | 0.538 | 3.56266 |
| 2.5 | 25.0 | 0.629 | 2.27736 |
| 5.0 | 50.0 | 0.829 | 0.42159 |

**Table S6.** Fraction affected (Fa) and CI values calculated for combination of MomC + SCH772984

| **MomC [µM]** | **SCH772984 [µM]** | **Fa** | **CI value** |
| --- | --- | --- | --- |
| 0.625 | 1.25 | 0.758 | 0.05916 |
| 1.25 | 2.5 | 0.764 | 0.10531 |
| 2.5 | 5.0 | 0.756 | 0.24592 |
| 5.0 | 10.0 | 0.872 | 0.03044 |
| 0.625 | 0.625 | 0.706 | 0.15118 |
| 1.25 | 1.25 | 0.707 | 0.29725 |
| 2.5 | 2.5 | 0.738 | 0.34418 |
| 5.0 | 5.0 | 0.843 | 0.07054 |

##

**Table S7.** Fraction affected (Fa) and CI values calculated for combination of MomC + enzalutamide

| **Enzalutamide [µM]** | **MomC [µM]** | **Fa** | **CI value** |
| --- | --- | --- | --- |
| 12.5 | 0.125 | 0.186 | 1.38397 |
| 25.0 | 0.25 | 0.348 | 1.57860 |
| 3.125 | 0.125 | 0.064 | 1.82108 |
| 6.25 | 0.25 | 0.197 | 1.77127 |
| 12.5 | 0.5 | 0.796 | 0.94072 |
| 3.125 | 0.125 | 0.079 | 1.58977 |
| 6.25 | 0.25 | 0.189 | 1.81996 |
| 12.5 | 0.5 | 0.757 | 1.04121 |

## Original Western blotting files


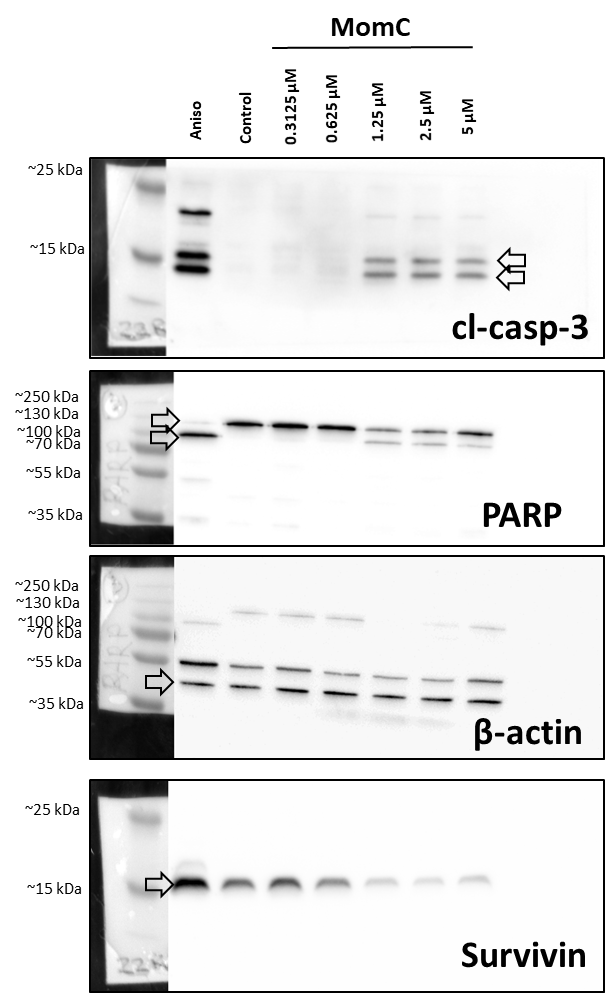


### **Figure 1S.** Original files for Figure 2a (Western blotting).


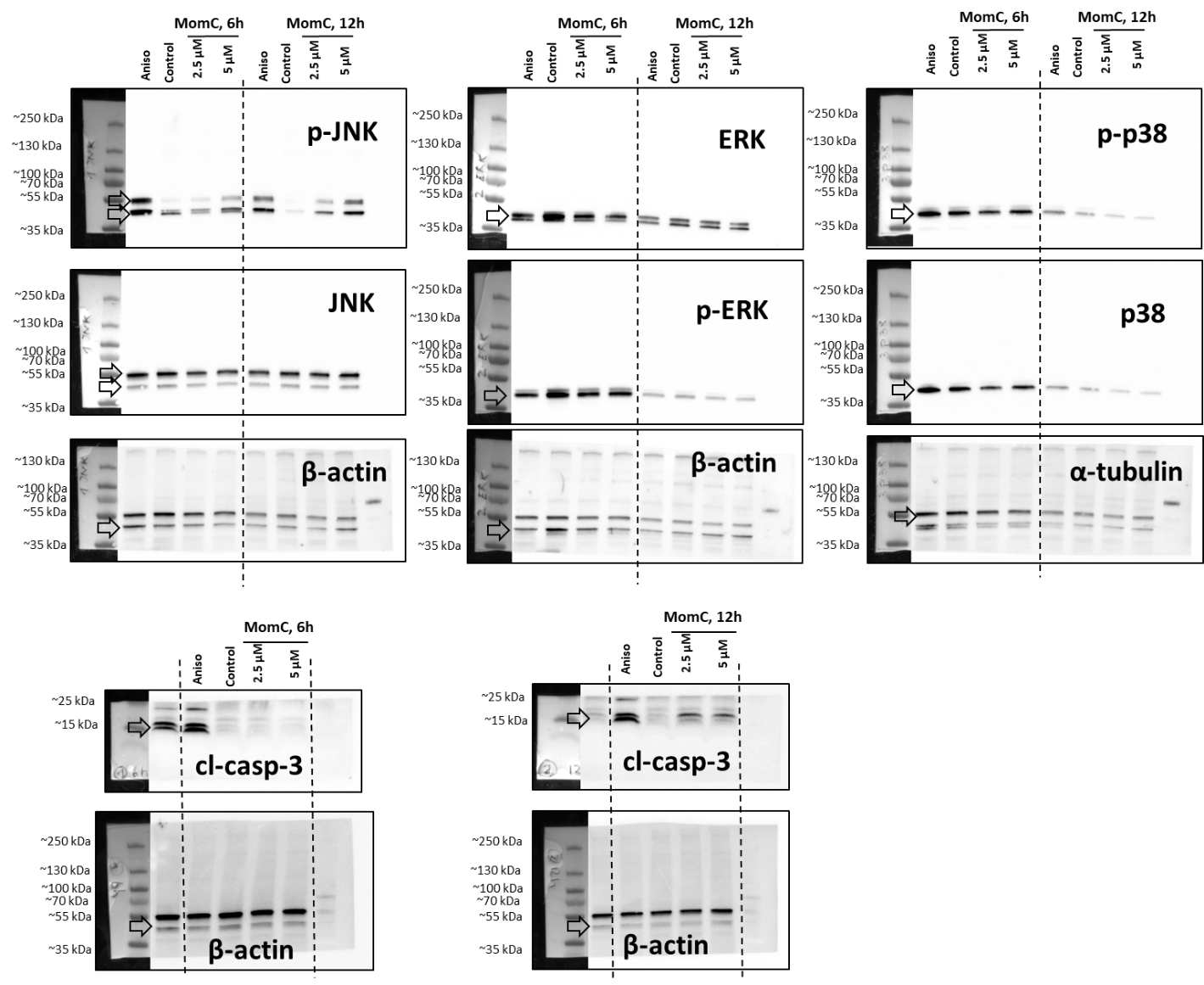


### **Figure 2S.** Original files for Figure 4a (Western blotting).


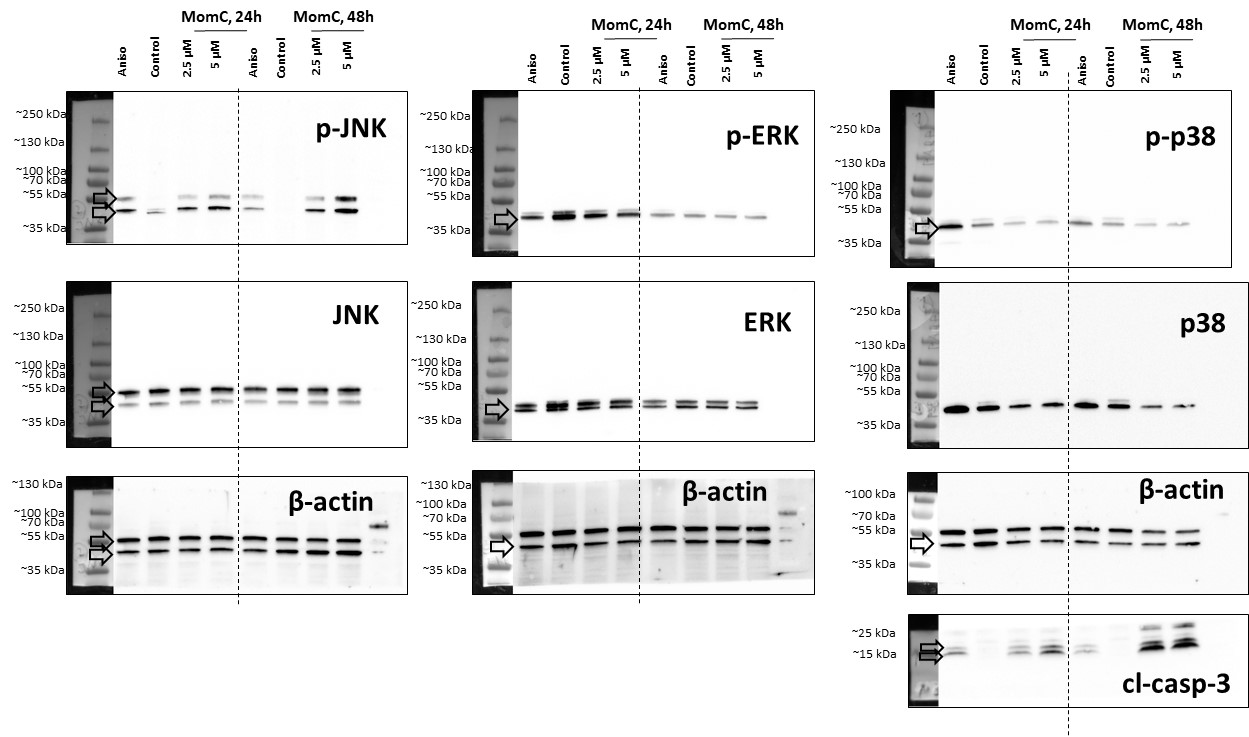


### **Figure 2S (continuation).** Original files for Figure 4a (Western blotting).


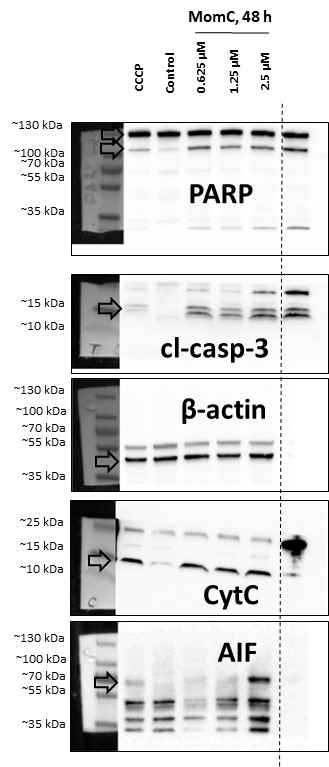


### **Figure 3S.** Original files for Figure 5c (Western blotting).
